# Safety profile of chlorhexidine and povidone-iodine in rectal mucosa cleansing during prostate biopsy

**DOI:** 10.3389/fruro.2023.1176965

**Published:** 2023-06-29

**Authors:** Adriana M. Pedraza, Jeffer David Álvarez Villarraga, María Alejandra Zapata Copete, Dhruti Patel, Herney Andrés García-Perdomo

**Affiliations:** ^1^ Desa, Rafael Uribe Uribe Clinic, Cali, Colombia; ^2^ Icahn School of Medicine at Mount Sinai, New York, NY, United States; ^3^ Department of Urology, Universidad Libre, Cali, Colombia; ^4^ UROGIV Investigation Group, Universidad del Valle, Cali, Colombia; ^5^ ICESI University, Cali, Colombia; ^6^ School of Medicine Pontificia Universidad Javeriana, Cali, Colombia; ^7^ Division of Urology/Urooncology, Department of Surgery, School of Medicine, Universidad del Valle, Cali, Colombia

**Keywords:** prostate biopsy, iodine povidone, chlorhexidine, rectal antisepsis, transrectal ultrasound

## Abstract

**Objective:**

To evaluate the use of rectal mucosal cleansings before transrectal ultrasound-guided prostate biopsy with a transrectal approach, comparing the safety profile of chlorhexidine and povidone-iodine.

**Methods:**

We conducted a retrospective analysis of our prospectively maintained database between August 2019 to September 2020 in a high-volume hospital in Cali, Colombia. 428 consecutive patients who underwent TRUS-PB with a transrectal approach were included in this study. 117 patients received povidone-iodine and 311 patients received chlorhexidine for rectal mucosa cleansings. After the procedure, we conducted telephone follow-ups at 48 hours, 7 days, and 30 days. The complications were registered in our database. Analysis was performed using STATA 15.

**Results:**

There was a statistically significant increased risk of hematuria, urinary retention, and rectal bleeding in those patients exposed to Chlorhexidine (p <0.001, <0.001, and 0.01 respectively). We did not find any differences in sepsis (*p* 0.18) or urinary tract infection (*p* 0.77) rates between the groups. Rectal antisepsis with chlorhexidine significantly increased the risk of non-infectious complications.

**Conclusions:**

In terms of infectious complications, there were no differences between the use of povidone-iodine and chlorhexidine for rectal mucosal cleansing prior to TRUS-PB. Povidone iodine appeared to be a safer option, as it is associated with fewer risks of hematuria, rectal bleeding, and urine retention.

## Introduction

Approximately two million prostate biopsies are conducted each year in the United States and Europe (1, 2). The upsurge of fluoroquinolone-resistant rectal bacteria has been linked to an increase in sepsis rates following transrectal (TR) prostate biopsies (1, 3). Furthermore, the latest guidelines of the European Association of Urology (EAU), recommend a transperineal approach supported by a 1a level of evidence (4). While the advantages of this approach are proven, its adoption has been restricted by the need for general anesthesia in most institutions and insufficient training on the technique. Therefore, numerous preventive methods against infectious complications have been established. These strategies include culture-based prophylaxis and the use of rectal povidone-iodine to reduce bacterial load.

A recent meta-analysis suggested that rectal preparation with povidone-iodine is an effective nonantibiotic technique for reducing the risk of infection and hospitalization after prostate biopsy when a transrectal, as opposed to a transperineal route, is utilized (5).

The active ingredient in the povidone-iodine solution polyvinylpyrrolidone (PVP), loses potency when applied to wet surfaces. Due to the rectal cavity’s exposure to mucus and feces, this solution may not have its maximal effect (6). On the other hand, chlorhexidine, in its alcohol form, could be more effective as it has high bioavailability in the mucosa and skin. Authors recommend chlorhexidine as the first choice in surgical procedures of different kinds (7). Despite chlorhexidine’s favorable reputation in surgical settings, there are no studies comparing the efficacy of the povidone-iodine enema to that of chlorhexidine in transrectal ultrasound-guided biopsies.

This study aimed to evaluate the use of rectal mucosal cleansings before Transrectal Ultrasound-guided Prostate Biopsy (TRUS-PB) with a transrectal approach, comparing the safety profile of chlorhexidine and povidone-iodine.

## Methods

### Study population

We conducted a retrospective analysis of our prospectively maintained database between August 2019 to September 2020 in a high-volume hospital in Cali, Colombia. Overall, 428 consecutive patients who underwent TRUS-PB with a transrectal approach were included in this study. Indications for prostate biopsy included an increased prostate-specific antigen (PSA), abnormal digital rectal examination (DRE), or evidence of Prostate Imaging-Reporting and Data System (PI-RADS) ≥ 3 on mpMRI (multiparametric Magnetic Resonance Imaging). All patients were administered a three-day course of antibiotics based on the results of the rectal swab, which identified whether the organisms were sensitive or resistant to quinolones. Furthermore, all patients had negative urine cultures. The implementation of targeted antibiotic prophylaxis involved the following protocol: patients who exhibited quinolone resistance in the rectal swab were prescribed fosfomycin, whereas those who were sensitive to quinolones but were deemed to be at high risk of carrying resistant organisms (diabetes, immunosuppression, recurrent urinary tract infections, recent antibiotic usage within six months, or healthcare workers) (8) were given amikacin 30 minutes before the procedure in addition to the standard 3-day course of quinolones. Conversely, patients who did not exhibit resistance on the rectal swab and who had no identified risk factors only received quinolones.

A polyethylene glycol (PEG) enema was prescribed to be used six hours before the procedure. Topical rectal antiseptic preparation using 10% povidone-iodine or 4% chlorhexidine without alcohol was applied at the discretion of the treating urologist. Prior to the TRUS-PB, the rectal mucosa was purged with 10 milliliters of the designated cleaning and given two minutes to act. All the biopsies were done by an experienced urologist (JDAV).

TRUS-PB was performed on the left decubitus position. A transrectal probe (endocavity biplane 8848 - BK Medical) was introduced after 2% lidocaine gel was instilled into the rectum. Then, 10 milliliters of 2% lidocaine were injected into the junction between the prostate and seminal vesicles using a 22-gauge needle. Following the conventional transrectal 12-core biopsy, cognitive-targeted biopsies were performed (3 to 4 additional cores per target) in those patients with abnormal mpMRI.

### Variables and definitions

Baseline information was gathered concerning age, PSA, and DRE. To follow up on the outcomes of the TRUS-PB, a dedicated nurse carried out patient-directed phone calls and chart reviews at specified intervals - 48 hours, 7 days, and 30 days after the procedure. Information was captured on a variety of complications post-procedure, including hematuria, urosepsis, urinary tract infections (UTI), rectal bleeding, hematospermia, acute urine retention (AUR), and mortality. Pathology results were extracted from the clinic’s database using the patient identifying number.

UTI was defined as lower urinary tract symptoms and a positive urine culture after the biopsy, whereas urosepsis was defined as UTI with organ dysfunction as per the third International Consensus definition for sepsis and septic Shock (9).

This study was approved by the institutional review board.

### Statistical analysis

Descriptive statistics were used to summarize the characteristics of the cohorts. T-tests and chi-square tests were used for continuous and categorical variables, respectively. After performing univariate binary logistic regression analyses, variables identified to be significantly associated with complications were included in a multivariable binary logistic regression analysis. Correlations among variables were determined using multivariate analysis. A p-value <0.05 was considered significant. We described the results with the odds Ratio (OR) and the corresponding 95% confidence interval (95%CI). Statistical analyses were performed using STATA 15.

## Results

A transrectal approach was used to perform TRUS-PB on 478 patients who met the inclusion criteria. However, 50 patients were lost to follow-up due to reasons such as a lack of interest in the study or communication barriers. Therefore, the analysis was conducted on a total of 428 patients. **Table 1** summarizes their baseline characteristics.

**Table 1:** Population Characteristics according to cleansing used.

	Chlorhexidine (N=311)	Povidone-iodine (N=117)	P value
Age			
*Median (Range)*	67 (31-92)	67 (44-82)	0.40
PSA			
*Median (Range)*	9.44 (0.3-100)	8.90 (1.4-100)	0.17
MRI			
*Yes*	15 (4.8)	10 (8.5)	0.14
*No*	296 (95.2)	107 (91.5)	
Rectal Examination, n (%)			
*Normal*	242 (77.8)	88 (75.2)	0.56
*Abnormal*	69 (22.2)	29 (24.8)	
PI-RADS, n (%)			
*1*	0 (0)	1 (0.8)	0.34
*2*	1 (0.3)	2 (1.7)	
*3*	2 (0.6)	0 (0)	
*4*	6 (1.9)	2 (1.7)	
*5*	6 (1.9)	6 (5.1)	
*NA*	296 (95.3)	106 (90.7)	
Pathology, n (%)			
*Negative*	189 (60.8)	61 (52.1)	<0.001
*Acinar adenocarcinoma*	115 (37)	46 (39.3)	
*Atypical small acinar proliferation*	5 (1.6)	10 (8.6)	
*Undifferentiated adenocarcinoma*	1 (0.3)	0 (0)	
*NA*	1 (0.3)	0 (0)	
**ISUP Gleason grading system, n (%)**			0.14
*Grade 1*	35 (11.2)	20 (17.1)	
*Grade 2*	52 (16.7)	13 (11.1)	
*Grade 3*	13 (4.2)	3 (2.6)	
*Grade 4*	12 (3.9)	8 (6.8)	
*Grade 5*	3 (1)	2 (1.7)	
*NA*	196 (63)	71 (60.7)	
Complications, n (%)			
*Yes*	209 (67.2)	30 (25.6)	**<0.001**
*No*	102 (32.8)	87 (74.4)	
**Number of cores, mean (SD)**	12.5 (1.3)	12.3 (0.9)	0.26

AUR, Acute Urinary Retention; UTI, Urinary Tract Infection; NA, not applicable.

Rectal chlorhexidine was administered to 311 patients, while 117 received rectal povidone-iodine. No significant differences in the baseline characteristics between the two groups were found. After the TRUS-PB, 167 patients (55.8%) reported complications. Hematuria was the most common one, followed by AUR, rectal bleeding, hematospermia, UTI, and sepsis (**Table 2**).

**Table 2:** Complications according to cleansing used.

	Chlorhexidine n= 209 (%)	Povidone-Iodine n= 30 (%)	*P* value
**Hematuria**	89 (42.6)	12 (40)	**<0.001**
**AUR**	50 (23.9)	2 (6.7)	**<0.001**
**Rectal Bleeding**	38 (18.1)	5 (16.7)	**0.015**
**Hematospermia**	19 (9.1)	4 (13.3)	0.34
**UTI**	11 (5.3)	5 (16.7)	0.77
**Sepsis**	1 (0.5)	2 (6.6)	0.18
**Mortality**	1 (0.5)	0 (0)	0.53

AUR, Acute Urinary Retention; UTI, Urinary Tract Infection.

Compared to povidone-iodine, patients who were exposed to chlorhexidine experienced a higher rate of complications (67.2% vs. 25.6%) with a statistically significant increased risk of hematuria, AUR, and rectal bleeding (p<0.001, <0.001, and 0.01, respectively). Overall, 6.9% of the patients with hematuria (7 out of 101) needed a Foley catheter insertion and continuous bladder irrigation. Specifically, 2 patients (1.9%) in the povidone-iodine group and 5 patients (4.9%) in the chlorhexidine group. Nonetheless, no interventions in the operating room were necessary for any of these cases.

Our analysis revealed no statistically significant differences in the incidence of urosepsis (p=0.18), UTI (p=0.77), or mortality (p=0.53) between the chlorhexidine and povidone-iodine groups. The occurrence of one case of sepsis in the chlorhexidine group is noteworthy as it required admission to the intensive care unit and eventually resulted in mortality. The presence of multiple pre-existing comorbidities in that specific patient may have explained this outcome.

Our multivariate analysis revealed that the number of cores taken during prostate biopsy was independently associated with a higher risk of non-infectious complications after TRUS-PB (OR: 1.5; 95% CI: 1.3-1.8). On the contrary, the use of povidone-iodine was linked to an 80% lower risk of non-infections complications compared to the use of chlorhexidine (OR: 0.2; 95% CI: 0.1 - 0.3) (**Table 3**).

**Table 3:** Uni- and multivariable binary logistic regression analysis predicting risk of non-infectious complications after TRUS-PB.

	Univariate Analysis			Multivariate Analysis		
Covariate	OR	95% CI	p	OR	95% CI	p
Number of cores	1.5	1.3 -1.8	<0.001	1.5	1.3 -1.8	**<0.001**
Topical rectal antiseptic	0.2	0.1 - 0.3	<0.001	0.2	0.1 - 0.3	**<0.001**

AUR, Acute Urinary Retention; UTI, Urinary Tract Infection.

## Discussion

Infectious complications after TRUS-PB with a transrectal route range from 0.1% to 7.0%, with sepsis rates ranging from 0.3% to 3.1%, depending on the antibiotic prophylactic regimens and baseline antibiotic resistance (1, 10). Consequently, different strategies to decrease these complications have been described, including topical antiseptic agents in the rectal mucosa applied before the procedure. To the best of our knowledge, there are only two studies published using povidone-iodine and chlorhexidine separately in this scenario. Park et al. (11) demonstrated that antisepsis in the rectal mucosa decreases the bacterial load by 97.5% in the povidone-iodine group vs. 99.3% in the chlorhexidine group (*p* 0.03). Furthermore, Ergani et al. (12) reported a reduction in sepsis among patients treated with topical rectal antiseptics in a recent randomized controlled trial (p 0.01), with no significant differences in infective complications between povidone-iodine and chlorhexidine. Our own study aligns with these findings, as we observed no significant differences in rates of sepsis or UTIs between chlorhexidine and povidone-iodine (p 0.18 and 0.77, respectively).

Hematuria is a common and typically self-limiting complication following TRUS-PB, with incidence varying between 6.5% to 47.1% in different studies (13, 14). In our cohort, hematuria was the most frequent complication, occurring in 23.5% of cases. Our analysis also revealed a higher risk of hematuria in patients who received rectal chlorhexidine compared to povidone-iodine (p <0.001). Although the amount of chlorhexidine that reaches the urothelium during TRUS-PB is likely negligible, its use for cleansing has been associated with adverse effects in some studies. For instance, Harper et al. observed severe erosive cystitis on histologic examination in a high percentage of rats whose bladders were irrigated with aqueous solutions of chlorhexidine digluconate (15). Furthermore, another study demonstrated that although chlorhexidine bladder irrigation may reduce the occurrence of bacteriuria after transurethral procedures, it also resulted in intolerable levels of hematuria (16). In contrast, iodine povidone has been proposed as a feasible hemostatic agent in various surgical fields (17).

The incidence of rectal bleeding following TRUS-PB ranges from 1.3% to 45% (1, 10). Although massive rectal bleeding is rare, it can be life-threatening. In our study cohort, only 10% of patients experienced this complication, but most cases were associated with rectal antisepsis using chlorhexidine (p 0.01). We did not find any similar association published in the literature that correlates with this finding in our study.

Importantly, none of the cases required additional treatment due to this complication.

We also noted a higher prevalence of AUR in the group treated with chlorhexidine (p < 0.001). AUR is a rare complication that occurs soon after a prostate biopsy. It is more prevalent in procedures that involve taking a larger number of biopsy cores, particularly transperineal approaches with over 24 cores (10). AUR is known to occur in the range of 0.2% to 4.6% after TRUS-PB (18). However, we observed a higher incidence rate of 12% in our study, with only 7 out of 52 cases attributed to hematuria. Unfortunately, due to limitations in data collection, we were unable to identify a clear explanation for this finding. Nevertheless, we did observe that patients who experienced AUR were generally older, with an average age of 71, compared to the overall cohort’s average age of 67 years.

Strong evidence supports the use of transperineal prostate biopsy given the lower risk of infection. However, this approach may face delays in its widespread implementation, particularly in countries with transitioning or developing economies. Obstacles to the adoption of transperineal biopsies include the requirement for general anesthesia, although local anesthesia alternatives have been documented, insufficient access to necessary equipment, limited reimbursement, and inadequate training in the technique during residency. Therefore, efforts to decrease the risk of infection with transrectal approaches are still ongoing.

We acknowledge the limitations of our study. Firstly, the data was obtained through patient questioning rather than by observing clinical outcomes, as some patients were not followed up in the same institution due to health insurance contracts with other centers. Additionally, we could not assess the severity of hematuria at presentation in all cases, hospital admissions related to this complication, or anticoagulation use in this cohort. Despite these limitations, to our knowledge, this is the first study to examine the safety profile of antiseptics on the rectal mucosa during TRUS-PB.

In summary, our study found no significant differences in terms of infective complications between the use of povidone-iodine and chlorhexidine for rectal mucosal cleansing prior to TRUS-PB. Povidone iodine appears to be the safer option, with fewer complications such as hematuria, rectal bleeding, and AUR. However, prospective randomized controlled trials are needed to validate these findings.

## Data availability statement

The original contributions presented in the study are included in the article/supplementary material. Further inquiries can be directed to the corresponding author.

## Ethics statement

The studies involving human participants were reviewed and approved by Rafael Uribe Uribe Hospital committee. The patients/participants provided their written informed consent to participate in this study.

## Author contributions

JÁ, HG, and AP contributed to the conception and design of the study. MZ organized the database. AP and MZ performed the statistical analysis. JÁ, AP, HG, and DP wrote the manuscript. All authors contributed to the article and approved the submitted version.
